# Minimal Active Space for Diradicals Using Multistate Density Functional Theory

**DOI:** 10.3390/molecules27113466

**Published:** 2022-05-27

**Authors:** Jingting Han, Ruoqi Zhao, Yujie Guo, Zexing Qu, Jiali Gao

**Affiliations:** 1 Institute of Theoretical Chemistry, College of Chemistry, Jilin University, Changchun 130023, China; hanjt16@mails.jlu.edu.cn (J.H.); zhaorq1214@mails.jlu.edu.cn (R.Z.); yjguo20@mails.jlu.edu.cn (Y.G.); 2 Institute of Systems and Physical Biology, Shenzhen Bay Laboratory, Shenzhen 518055, China; 3 Beijing (Peking) University Shenzhen Graduate School, Shenzhen 518055, China; 4 Department of Chemistry and Supercomputing Institute, University of Minnesota, Minneapolis, MN 55455, USA

**Keywords:** minimal active space (MAS), MSDFT, diradicals, singlet-triplet-energy gap

## Abstract

This work explores the electronic structure as well as the reactivity of singlet diradicals, making use of multistate density functional theory (MSDFT). In particular, we show that a minimal active space of two electrons in two orbitals is adequate to treat the relative energies of the singlet and triplet adiabatic ground state as well as the first singlet excited state in many cases. This is plausible because dynamic correlation is included in the first place in the optimization of orbitals in each determinant state via block-localized Kohn–Sham density functional theory. In addition, molecular fragment, i.e., block-localized Kohn–Sham orbitals, are optimized separately for each determinant, providing a variational diabatic representation of valence bond-like states, which are subsequently used in nonorthogonal state interactions (NOSIs). The computational procedure and its performance are illustrated on some prototypical diradical species. It is shown that NOSI calculations in MSDFT can be used to model bond dissociation and hydrogen-atom transfer reactions, employing a minimal number of configuration state functions as the basis states. For p- and s-types of diradicals, the closed-shell diradicals are found to be more reactive than the open-shell ones due to a larger diabatic coupling with the final product state. Such a diabatic representation may be useful to define reaction coordinates for electron transfer, proton transfer and coupled electron and proton transfer reactions in condensed-phase simulations.

## 1. Introduction

“It is hard to imagine complex chemistry without diradicals”, said Hoffmann and coworkers [1]. Diradicals are reactive species and have been extensively studied both theoretically and experimentally [1,2,3,4,5]. The spin coupling between two unpaired electrons results in a singlet state and a low-energy triplet state [6,7,8,9,10]. Both states, having an *M_S_* = 0, are of multiconfigurational character that cannot be adequately treated by Kohn–Sham density functional theory (KS-DFT) [6,7,8]. However, the energy-degenerate *M_S_* = ±1 spin multiplets of the triplet state can be represented by a single determinant using KS-DFT [6,7]. Furthermore, electron correlation plays an important role between the two closed-shell configurations in a two-electron and two-orbital active space, which ultimately determines the relative energies of the singlet and triplet ground states [9,10]. Consequently, the need to use a multiconfigurational method to treat open-shell diradicals poses a significant challenge to KS-DFT, and often a broken-symmetry approach is used to estimate the singlet–triplet-energy gap [11,12,13]. On the other hand, the difficulties of KS-DFT can be easily overcome using multiconfiguration self-consistent-field (MCSCF) methods in wave function theory (WFT), such as the complete-active-space self-consistent-field (CASSCF) approach, but, in this case, it is necessary to use a large active space, followed by including corrections for dynamic correlation to obtain quantitative results [14,15,16,17]. In this study, we present a multistate density functional theory (MSDFT) [18], in which a minimal active space (MAS) is sufficient to describe biradical species and to determine the singlet–triplet-energy gaps. 

MSDFT makes use of a Hamiltonian *matrix functional* of the multistate density D(r), whose optimization yields the excited-state energies and densities (provided that an approximate universal correlation matrix functional is available) [18,19,20,21,22]. Previous studies show that MSDFT can be a practical procedure to treat the ground and excited states on an equal footing [18,19,20,21,22]. Our goal is to use a minimal number of charge, spin or excitation-localized configurations, having valence-bond-like characters to represent charge transfer (CT) and excited configurations of molecular complexes [23,24,25,26,27]. A convenient approximation, in the spirit of configuration interaction (CI) in WFT, is to optimize the individual basis states in the active space that is sufficient to treat a given problem. Such a constrained KS-DFT optimization can be accomplished by fragment block-localization or by targeted orbital optimization techniques [20,23,24]. Consequently, orbitals in different determinant functions are nonorthogonal. Since these Slater determinants are used to represent the multistate density D(r) for the real interacting system, this procedure is called nonorthogonal state interaction (NOSI) [23,24,25,26] to distinguish it from a nonorthogonal configuration interaction (NOCI) in which the dynamic correlation is absent [21,22]. Formally, the multistate matrix density D(r) corresponds to that derived from the corresponding contracted states of the full configuration interaction (FCI) wave function through singular value decomposition [20]. Then, adiabatic state energies are obtained by diagonalization of the Hamiltonian matrix, which yields vectors that are also eigenfunctions of **S**^2^ [27]. NOSI may be considered as one variant of the dynamic-then-static ansatz described by Liu and coworkers [28]. Moreover, diabatic states can be constructed within MSDFT framework [23,29,30].

The reactivities of singlet diradicals have been extensively studied in the past [31,32,33,34] and lucidly summarized [1]. Here, we present the results from NOSI calculations on a set of prototypical examples involving diradicals, and make comparison with those that have been extensively studied to demonstrate the performance of MSDFT [35,36,37]. In the following, we first describe the theoretical background and computational details. In each case, we outline the procedure for constructing a MAS for the question of interest. Then, we present the results and discussion on singlet- and triplet-energy gaps for a series of compounds and the hydrogen abstraction reaction of SiH_4_ by a p-type (cyclobutadiene) and an s-type (*p*-benzyne) diradical.

## 2. Theoretical Background

The matrix element of the Hamiltonian matrix functional H[D(r)] of the multistate density D(r) in the subspace spanned by *N* states, ℛ^N^∈ℋ, is given by [18,20,23]

(1) $$H_{AB}[D(r)]=T_{AB}+E_{AB}^{Hx}[D_{AB}(r)]+\int drD_{AB}(r)v_{ext}(r)+E_{AB}^{xc}[D_{AB}(r)],$$

where the terms on the right-hand side of the equation are, respectively, the kinetic energy, Coulomb (Hartree) and exchange energy, the external potential energy, and the exchange-correlation energy for the interactions between states *A* and *B*. If A=B, Equation (1) is equivalent to KS-DFT for the density $D_{AA}(r)=\rho_{A}(r)$ represented by the determinant $\Phi_{A}$. However, for A≠B, each term is a functional of the transition density D(r). Both state and transition densities are related to the one-particle density matrix $P_{AB}$ (below); it is important to emphasize that the matrix density D(r) is not to be confused with $P_{AB}$, which is not a function of **r**.

(2) $$D_{AB}(r)=\sum_{\mu \nu }^{m}\left|\chi_{\mu }(r\right)>{\left(P_{AB}\right)}_{\mu \nu }<\chi_{\nu }(r)|=\chi^{T}(r)P_{AB}\chi (r),$$

where χ(r) is a column vector of *m* atomic orbital basis functions, and the one-particle density matrix $P_{AA}$ (A=B) and transition density matrix $P_{AB}$ (A=B) for determinants $\Phi_{A}$ and $\Phi_{B}$ are related to the coefficient matrices $C^{A}$ and $C^{B}$ of occupied orbitals by

(3) $$P_{AB}=C^{B}{\left[{\left(C^{A}\right)}^{T}RC^{B}\right]}^{-1}{\left(C^{A}\right)}^{T},$$

with R being the overlap matrix of the basis functions [18,20].

For the molecular systems considered in this work, the external potential in Equation (1) is simply the nucleus-electron Coulomb energy, and the first three terms can be expressed together by

(4) $$\{T_{AB}+E_{AB}^{Hx}[D_{AB}]+\int drD_{AB}(r)v_{ext}(r)\}=Tr(P_{AB}h)+\frac{1}{2}Tr(P_{AB}GP_{AB}),$$

where **h** and G are standard matrices of one-electron integrals (kinetic and nuclear attraction integrals) and two-electron Coulomb-exchange integrals. For the last term of Equation (1), Kohn–Sham exchange-correlation functional can be used to approximate the diagonal element of the multistate matrix functional, $E_{AA}^{xc}[D_{AA}(r)]=E_{xc}^{KS}[D_{AA}(r)]$, but the off-diagonal elements are the transition density correlation functional (TDF), which does not exist in KS-DFT [20]. In special situations, such as the present singlet–triplet-state energy difference involving spin-pairing interactions between two unpaired electrons, the TDF energy can be obtained by enforcing the spin-multiplet degeneracy condition of the triplet states [24,25,26,29].

(5) $$E_{AB}^{xc}[D_{AB},\Phi_{A}^{↑↓},\Phi_{B}^{↓↑}]=E_{c}^{KS}[D_{T}(\Phi_{T}^{↑↑})]-E_{c}^{KS}[D_{AA}(\Phi_{A}^{↑↓})],$$

In Equation (5), $E_{c}^{KS}[\rho_{T}(\Phi_{T}^{↑↑})]$ and $E_{c}^{KS}[D_{AA}(\Phi_{A}^{↑↓})]$ are correlation energies computed using KS-DFT correlation functional for the triplet state and spin-contaminated configuration. The dynamic correlation energy contribution to the electronic coupling, $E_{AB}^{xc}[D_{AB},\Phi_{A}^{↑↓},\Phi_{B}^{↓↑}]$ between two spin-mixed determinants treated by KS-DFT ensures that the resulting triplet state with *M_S_* = 0 is degenerate with the *M_S_* = +1 component [25,26]. In turn, the pure singlet state energy is also determined. We note that the *M_S_* = +1 component of a triplet state can be adequately treated by KS-DFT with a single Slater determinant. Since the KS exchange-correlation functional leads to the exact ground-state energy, the TDF energy for diabatic electronic coupling in Equation (5) is exact, even though the specific functional form of TDF is unknown.

For non-spin-coupled determinants, we use the KS-DFT energy-weighted correlation energy to approximate the correlation energy of the TDF:

(6) $$E_{AB}^{xc}[D_{AB}(r)]=\frac{1}{2}\frac{H_{AB}^{NO}}{H_{A}^{HF}+H_{B}^{HF}}\left[E_{c}^{KS}\left(D_{A}(r)\right)+E_{c}^{KS}\left(D_{B}(r)\right)\right],$$

where $H_{AB}^{NO}=<\Phi_{A}|H|\Phi_{B}>$ is the nonorthogonal matrix element of the determinants $\Phi_{A}$ and $\Phi_{B}$, and the energies in the denominator are Hartree–Fock energies using KS orbitals.

The adiabatic eigenstate energies are obtained by diagonalizing the Hamiltonian matrix function H[D(r)]:

(7) $$E_{I}^{MS}[D(r)]=\sum_{A}^{}a_{AI}^{2}H_{AA}[D_{AA}]+\sum_{A\neq B}a_{AI}a_{BI}H_{AB}[D_{AB}],$$

where *I* denotes the *I*th adiabatic eigenstate, and *A* and *B* specify determinant functions {$\Phi_{A}$} representing the matrix density D(r). Equation (7) shows that $E_{I}^{MS}[D(r)]$ is an implicit functional of the eigenstate density $\rho_{I}(r)=\sum_{A}^{}a_{AI}^{2}D_{AA}(r)+\sum_{A\neq B}a_{AI}a_{BI}D_{AB}(r)$, which is not directly used as an input in a Kohn–Sham density functional approximation; a KS functional is optimized corresponding to the residual kinetic energy from a single determinant.

## 3. Computational Details

Molecular geometries were optimized using the Gaussian09 software with the M06-HF functional (guess = mix option was used in open-shell calculations) [38]. For comparison, we also performed CASSCF and CASPT2 calculations using the MolPro2012 package [39]. Dunning’s correlation consistent valence triple-zeta basis set (cc-pVTZ) was used unless specifically noted in the text [40].

In MSDFT calculations, the diagonal matrix elements of the Hamiltonian matrix functional is determined either by block-localized KS-DFT, in which effective valence bond-like configurations are represented by fragment block-localized orbitals [18], or by targeted optimization using a delta-SCF approach with a pre-selection transformation of the target orbitals [41]. For the off-diagonal elements, the transition density correlation functional (TDF) was obtained by applying the spin multiplet degeneracy constraint for spin coupling interactions (Equation (5)), or by a determinant-coupling weighted correlation energy for other situations (Equation (6)). The latter scales similarly to that with an overlap weight [20,23]. All determinant states were optimized using unrestricted KS-DFT, constrained either by fragment block-localization [23] or by a non-aufbau configuration with specific target orbital occupations [41], i.e., delta-SCF optimization. Additional computational details are provided in the text and in Supporting Information. MSDFT calculations were performed using a program interfaced with a locally modified GAMESS program for integral evaluations [42].

## 4. Results and Discussion

### 4.1. Potential Energy Curves of Hydrogen Molecule

We first illustrated the computational procedure of the MSDFT-NOSI method with a minimal active space (MAS) by computing the potential energy curves for the lowest 4 states in the *M_S_* = 0 manifold (three singlet and one triplet) of a hydrogen molecule (H_2_) [1,43,44]. The minimal representation of these four states includes two doubly occupied closed-shell (CS) configurations, and a pair of singly occupied open-shell (OS) configurations by placing two electrons in the two lowest-energy orbitals. Thus, the state densities of the subspace ℛ^4^ for the lowest four states of H_2_ are represented by the following configuration state functions:

(8) $$\Theta_{\mathrm{CS}}^{20}=\phi_{\mathrm{CS}}^{20}=\hat{A}(\chi_{H}^{\alpha }\chi_{H}^{\beta }),$$

(9) $$\Theta_{\mathrm{CS}}^{02}=\phi_{\mathrm{CS}}^{02}=\hat{A}(\chi_{L}^{\alpha }\chi_{L}^{\beta }),$$

(10) $$\Theta_{\mathrm{OS}}^{S}=\frac{1}{\sqrt{2}}\{\phi_{\mathrm{OS}}^{\alpha \beta }-\phi_{\mathrm{OS}}^{\beta \alpha }\},$$

(11) $$\Theta_{\mathrm{OS}}^{T}=\frac{1}{\sqrt{2}}\{\phi_{\mathrm{OS}}^{\alpha \beta }+\phi_{\mathrm{OS}}^{\beta \alpha }\},$$

where $\hat{A}$ is the antisymmetry operator, $\left\{\phi_{x}^{y}\right\}$ are Slater determinant functions, and $\left\{\Theta_{x}^{y}\right\}$ are configuration state functions (CSFs), in which spin configurations are indicated by the superscripts (y = 0, 2, α, β and S/T), and the way of orbital occupation is denoted by the subscripts (x = CS, OS) with the OS states defined by linear combinations of $\phi_{\mathrm{OS}}^{\alpha \beta }=\hat{A}(\chi_{H}^{\alpha }\chi_{L}^{\beta })$ and $\phi_{\mathrm{OS}}^{\beta \alpha }=\hat{A}(\chi_{H}^{\beta }\chi_{L}^{\alpha })$. In Equations (8) and (9), $\chi_{H}$ and $\chi_{L}$ are, respectively, the highest occupied molecular orbital (HOMO) and the lowest unoccupied molecular orbital (LUMO). Note that although the same symbols $\chi_{H}$ and $\chi_{L}$ are used to denote Kohn–Sham orbitals, they are generally different in different determinants and are nonorthogonal.

Because of spin symmetry, the triplet state $\Theta_{\mathrm{OS}}^{T}$ (Equation (11)) is orthogonal to the singlet states (Equations (8)–(10)). Thus, the matrix functional H[D(r)] of MSDFT is block diagonal. However, in the basis of spin-mixed determinants $\left\{\phi_{x}^{y}\right\}$ that are used in the present NOSI calculation, all four configurations must be included. Importantly, since $\Theta_{\mathrm{OS}}^{S}$ and $\Theta_{\mathrm{OS}}^{T}$ result from spin coupling interactions, the triplet-state energy degeneracy uniquely defines the value of the off-diagonal correlation matrix element, the TDF energy, between $\phi_{\mathrm{OS}}^{\alpha \beta }$ and $\phi_{\mathrm{OS}}^{\beta \alpha }$[25,26,29,45].

The four adiabatic states are obtained by diagonalizing the matrix functional H[D(r)] to yield

(12) $$\Psi_{1u}^{S_{0}}=a_{1u}^{20}\Theta_{\mathrm{CS}}^{20}-a_{1u}^{02}\Theta_{\mathrm{CS}}^{02}$$

(13) $$\Psi_{1g}^{S_{1}}=a_{1g}^{20}\Theta_{\mathrm{CS}}^{20}+a_{1g}^{20}\Theta_{\mathrm{CS}}^{02}+a_{1g}^{S}\Theta_{\mathrm{OS}}^{S}$$

(14) $$\Psi_{2g}^{S_{2}}=a_{2g}^{20}\Theta_{\mathrm{CS}}^{20}+a_{2g}^{20}\Theta_{\mathrm{CS}}^{02}-a_{2g}^{S}\Theta_{\mathrm{OS}}^{S}$$

(15) $$\Psi_{1u}^{T}=\Theta_{\mathrm{OS}}^{T}$$

where Equations (12)–(15) are the four lowest adiabatic states of H_2_, and $\left\{a_{x}^{y}\right\}$ are the coefficients of CSFs. Note that all adiabatic states are of a multiconfigurational character, including the closed-shell states, although $\Theta_{\mathrm{CS}}^{20}$ makes the predominant contribution to the ground state near bonding distances. 

**Figure 1:**
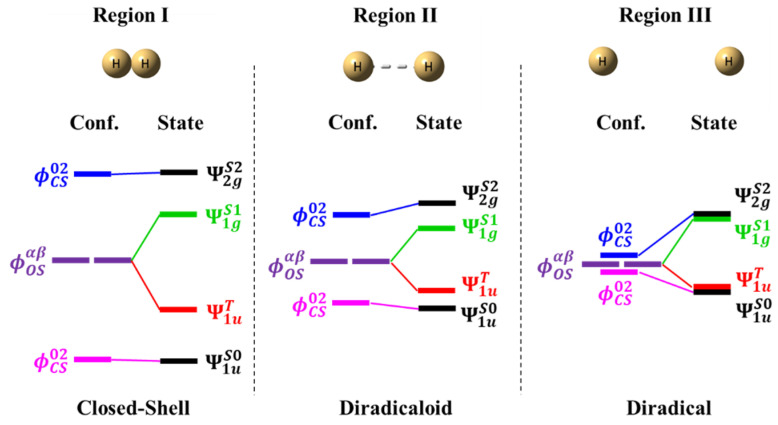
Schematic illustration of the relative energies of four diabatic (basis states) and adiabatic states of H_2_, varying from a predominantly closed-shell (**I**) configuration, to diradicaloid (**II**), ending at a diradical state (**III**) at infinite separation.

**Figure 2:**
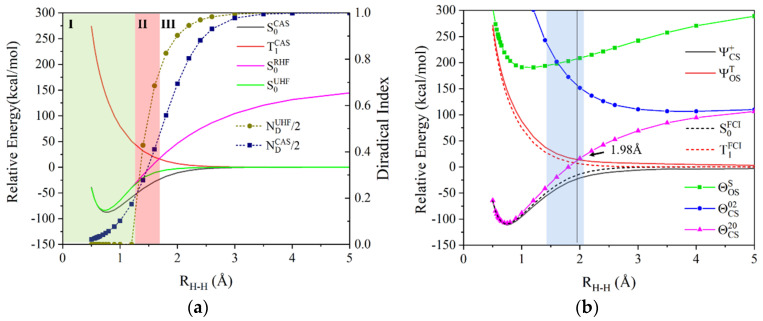
(**a**) Potential energy curves of the singlet ground and first triplet states, S0 and T1, and diradical index N_D_/2 for H_2_ determined by RHF, UHF and CASSCF methods. (**b**) Computed potential energy curves for the configuration state functions (colored symbols) and the lowest singlet and triplet ground states using MSDFT-NOSI (solid curves) along with the FCI results (dotted curves) for comparison. All calculations were performed with the cc-pVQZ basis set and the M06-HF functional was used in MSDFT calculations.

Figure 1 illustrates that the ground-state potential energy curve of H_2_ along the bond-distance coordinate can be roughly divided into three regions, corresponding to closed-shell (I), diradicaloid (II) and diradical (III) [1]. In region I, at the bonding distance, the energy of the $\Theta_{\mathrm{CS}}^{20}$ state is below the T_1_ state ($\Psi_{1u}^{T}$), contributing dominantly to the singlet ground state $\Psi_{1u}^{S_{0}}$ (Figure 2a). In region II, the $\Psi_{1u}^{S_{0}}$ singlet ground state is characterized as a diradicaloid [1], which has an increasing amount of multireference character with a large and rapidly increasing diradical index in Figure 2a [9]. Finally, in region III, the $\Theta_{\mathrm{CS}}^{20}=\phi_{\mathrm{CS}}^{20}$ configuration (Equation (8)) has a higher energy than that of the triplet state (Figure 1), but $\Psi_{1u}^{S_{0}}$ is still below the triplet state as a diradical species. In this region, the singlet and triplet ground states are nearly degenerate (degenerate at infinite separation). Here, the singlet state can be equivalently viewed as a multiconfiguration combination of two CS configurations (Equation (12)) and two localized open-shell diradicals as in valence bond (VB) representation. Here, the triplet state results from the combination of two spin-mixed OS determinants (Equation (15)). Nevertheless, the geometry at which the energies of the closed-shell CSF $\Theta_{\mathrm{CS}}^{20}$ and the triplet-state $\Psi_{1u}^{T}$ switch order may be used as the transition point from the closed-shell diradicaloid bonding character to a singlet diradical species.

Figure 2b illustrates the diabatic potential energy cures of the singlet CSFs (Equations (8)–(11)), and the lowest singlet $\Psi_{1u}^{S_{0}}$ and triplet $\Psi_{1u}^{T}$ adiabatic states for H_2_ from MSDFT-NOSI calculations along with the FCI results (dotted curves). The agreement between MSDFT and FCI results is good, although the relative energy between the singlet and triplet states from MSDFT-NOSI is somewhat greater than that of FCI, suggesting that electronic coupling between the two CS configurations may be overestimated. Significantly, the correct trend of the potential energy curve for H-H dissociation is obtained by MSDFT-NOSI through state interaction between the incorrect dissociation behavior of the spin-mixed determinants ($\Theta_{\mathrm{CS}}^{20}$ and $\Theta_{\mathrm{CS}}^{02}$). The area in blue between 1.45 Å and 2.2 Å in Figure 2b corresponds to region II in Figure 2a, which extends to longer bond distances than the latter (CASSCF) calculations without including the dynamic correlation. Figure 2 highlights the gradual transition in bonding character from a closed-shell configuration to a diradical state. The crossing point between the $\Theta_{\mathrm{CS}}^{20}$ diabatic state and the triplet state $\Psi_{1u}^{T}$ is located at 1.98 Å from MSDFT, consistent with the earlier studies [1,46].

### 4.2. Singlet–Triplet-Energy Gap

The singlet–triplet-energy splitting, $\Delta E_{ST}=E_{S}-E_{T}$, is an important property of diradicals [47,48,49,50]. Here, we summarize the results for three sets of compounds, including benzyne isomers, cyclobutadiene and polyacenes.

#### 4.2.1. Benzyne Isomers

Table 1 lists the computed singlet–triplet-energy gaps for the three benzyne isomers (meta, ortho and para) using DFT and WFT approaches [51] along with experimental values [52]. We found that methods that included both static and dynamic correlations clearly outperformed those that lacked either dynamic (CASSCF) or static correlation (UDFT) in comparison with experimental data. MSDFT employing the M06-HF functional and SF-CCSD, both of which included dynamic and strong correlations, had the lowest mean unsigned errors (MUEs), ranging from 0.5 to 0.7 kcal/mol. CASPT2 also yielded reasonable results with a somewhat larger MUE (1.3 kcal/mol) than MSDFT and SF-CCSD approaches. CASSCF systematically underestimated $\Delta E_{ST}$ for this series of compounds, having the largest unsigned errors (6.8 kcal/mol) among multiconfigurational methods. Interestingly, the use of unrestricted orbitals (UDFT) produced results of a similar quality as that of CASSCF calculations. It is interesting to note that the energies determined for *o*-benzyne were the same for RDFT and UDFT, strongly suggesting that a closed-shell configuration was the singlet ground state of this molecule [52]. On the other hand, *p*- and *m*-benzyne showed significant spin-contamination, evidenced by the computed S^2^ values (Table S5) and the large difference between $\Delta E_{ST}$ from UDFT (underestimate) and RDFT (overestimate). Clearly, static correlation plays an important role to adequately describe the singlet diradicals.

#### 4.2.2. Cyclobutadiene

**Figure 3:**
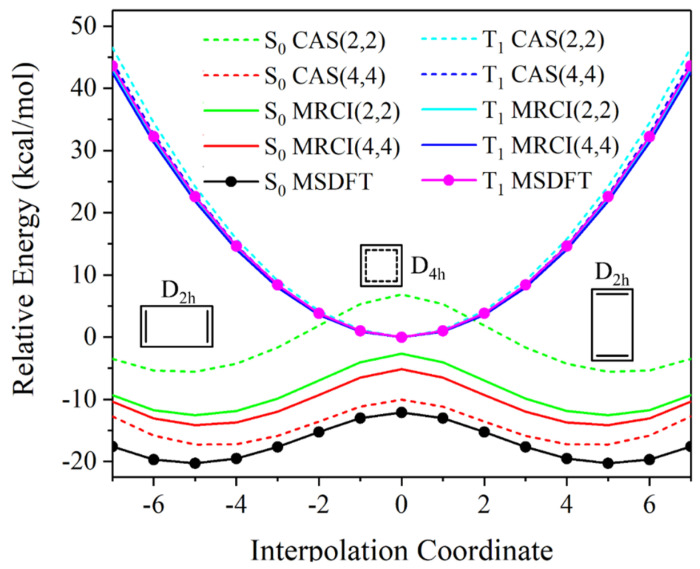
Computed potential energy curves for the ground-state singlet S_0_ and triplet T_1_ states of cyclobutadiene along the interpolated reaction coordinate between the two rectangular (D_2h_) geometries at a unitless value of ±5 through the transition state geometry (D_4h_). The cc-pVTZ basis set was used in all calculations. The energy of triplet state at the square geometry was set as the reference, since it has the least multiconfigurational character. The M06-HF functional was used in MSDFT calculations.

The change in $\Delta E_{ST}$ versus the geometry interconversion between the two rectangular structures were examined (Figure 3). The transition-state structure at the squared structure at D_4h_ symmetry on the singlet-state potential energy surface was a minimum of the triplet state [53]. At this geometry, the lowest singlet state corresponds to a combination of two doubly occupied (closed-shell) configurations [54,55], which can undergo Jahn–Teller distortion, leading to two rectangular geometries with D_2h_ symmetries [56]. The potential energy profiles determined with the CASSCF, multi-reference configuration interaction (MRCI) and MSDFT methods are shown in Figure 3 for the lowest singlet and triplet states. In Figure 3, the reaction coordinates for the interconversion between the two rectangular structures is interpolated to match the difference between the two rectangular sides (bond lengths). At the square geometry (D_4h_), the interpolation reaction coordinate is 0, and the two rectangular structures correspond to a unitless value of ±5. We used a minimum active space of two electrons in two orbitals (Equations (8)–(11)) both for MSDFT and CASSCF, whereas an active space of 4 electrons in 4 orbitals was also employed in the multireference MRCI calculations. However, we emphasized that a common set of orbitals were used both in CASSCF and in MRCI calculations, but nonorthogonal orbitals were adopted in the present MSDFT-NOSI.

Figure 3 shows that, at the optimal geometries of the rectangular minima, all methods produce the correct order in energy where the singlet state is below the triplet state. Interestingly, triplet-state energies are essentially the same for all methods, but there is significant variation in the energy of the singlet state among the different methods. In particular, the singlet state is above the triple at the D_4h_ geometry by CASSCF (2,2). The correct $\Delta E_{ST}$ difference is obtained from MRCI and MSDFT, both using a minimal active space of two electrons in two orbitals. This is confirmed further by the comparison with MRCI calculations using a larger active space of four electrons in four orbitals [57]. Interestingly, the correct $\Delta E_{ST}$ difference at the square geometry can also be achieved by expanding the active space to four electrons in four orbitals, even without perturbation correction (Figure 3). Clearly, the amount of dynamic correlation introduced by expanding the size of the active space is sufficient to recover the correct energy difference. The effect introduced by using a larger active space has been called spin-dynamic correlation in the literature [58,59,60]. Of all the methods examined, MSDFT-NOSI, employing M06-HF/cc-pVTZ, yields a somewhat large $\Delta E_{\mathrm{ST}}$ value at 23.2 kcal/mol. SF-EOM-CCSD results are found at 16 kcal/mol [57]. In closing this discussion, we note that Jahn–Teller effects are found in a variety of systems, for example, recent computational studies of gold nanoclusters that include 25 gold atoms in different oxidation states [61], a classical example of the compressed and elongated conformers resulting from ionization of benzene [20,25,62], and coronene radical cation [63].

#### 4.2.3. Polyacenes

**Figure 4:**
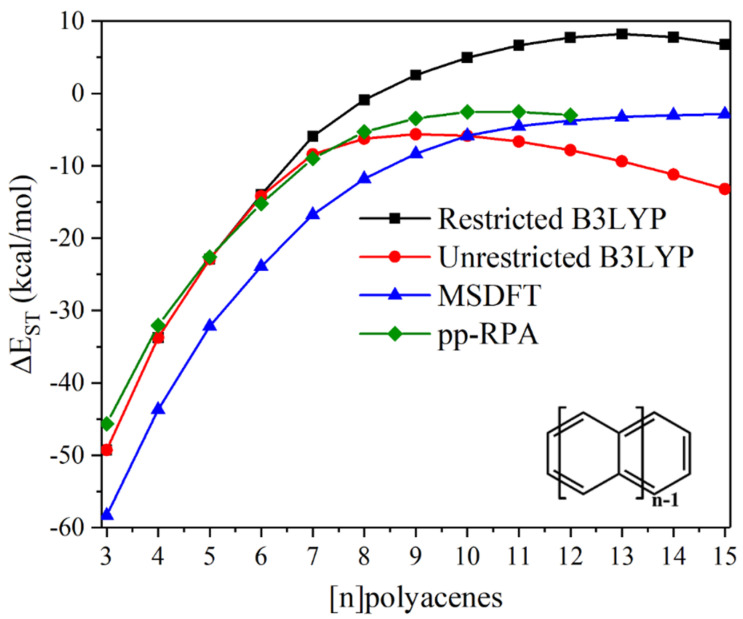
The adiabatic singlet-triplet-energy gaps $\Delta E_{\mathrm{ST}}$ in kcal/mol for the polyacene series. Restricted (RDFT) and unrestricted (UDFT) Kohn–Sham density functional theory and multistate density functional theory (MSDFT), employing the B3LYP functional and the 6-31G(d) basis set are shown, along with computational results from the pp-RPA approach [67] calculations.

The singlet–triplet-energy gaps in the series of polyacenes have been extensively studied experimentally and computationally [17,64,65,66,67,68]. Since there is a large number of calculations in the literature, we chose to use the work by Yang and coworkers for a comparison with the present results [67]. Shown in Figure 4 are the computed $\Delta E_{\mathrm{ST}}$ in the range of *n* = 3 to 15 rings, using both restricted and unrestricted KS-DFT (RDFT and UDFT) and MSDFT, employing the M06-HF functional and 6–31G(d) basis set [69], along with the computational results obtained by Yang and coworkers [67]. RDFT results incorrectly show an $\Delta E_{\mathrm{ST}}$ energy inversion between singlet and triplet states at *n* = 8, but the values both from UDFT and from MSDFT calculations indicate that the energies of the singlet state are uniformly below those of the triplet state in this series of compounds. This finding is consistent with a previous study that employed the density matrix renormalization group (DMRG) method, where the ground state remains to be singlet as the chain length increases with a finite singlet–triplet gap in the infinite chain limit (2–12 kcal/mol) [1,17]. The computed $\Delta E_{\mathrm{ST}}$ from UDFT exhibits a maximum at *n* = 9, in contrast to MSDFT results and DMRG studies. Interestingly, the particle–particle random phase approximation (pp-RPA) results seem to follow the UDFT trend [67], although at a much slower rate (Figure 4). Nevertheless, the difference in $\Delta E_{\mathrm{ST}}$ propagation between UDFT and RDFT calculations may indicate that there is a transition from a predominantly closed-shell diradicaloid configuration to a diradical state as the number of fused rings increases beyond 8. Figure 4 illustrates that MSDFT-NOSI with a minimal active space of [2,2] nonorthogonal configurations can adequately balance static and dynamic correlations through this transition.

### 4.3. Hydrogen-Atom Transfer Reactions

The hydrogen-atom transfer (HAT) reactions of SiH_4_ by the singlet diradicals of cyclobutadiene and para-benzyne may be formally considered as a concerted electron–proton transfer (CEPT) process [1], and we previously introduced a diabatic state representation of the different natures in electronic structure between HAT and CEPT using MSDFT [30]. Since these two reactions are known to be hydrogen atom abstraction, we restricted our discussion to the HAT diabatic states.

**Figure 5:**
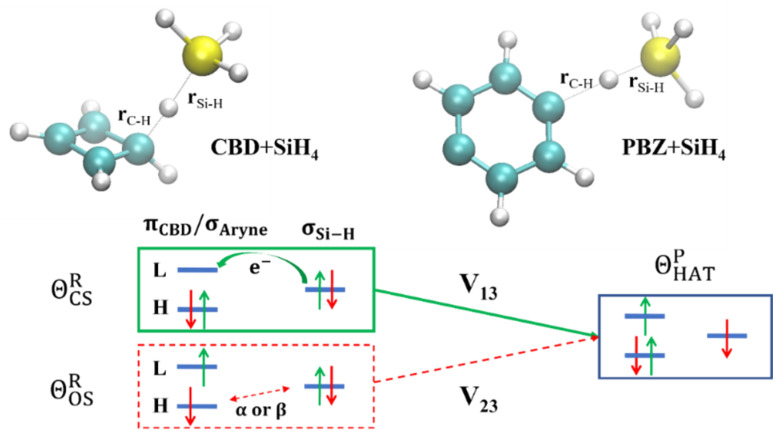
Depiction of the reactant diabatic states, representing the closed-shell (CS) and open-shell (OS) diradicals, and the product diabatic state for the hydrogen-atom transfer (HAT) reactions from SiH_4_ to cyclobutadiene (CBD) and *p*-benzyne (PBZ).

**Scheme 1:**
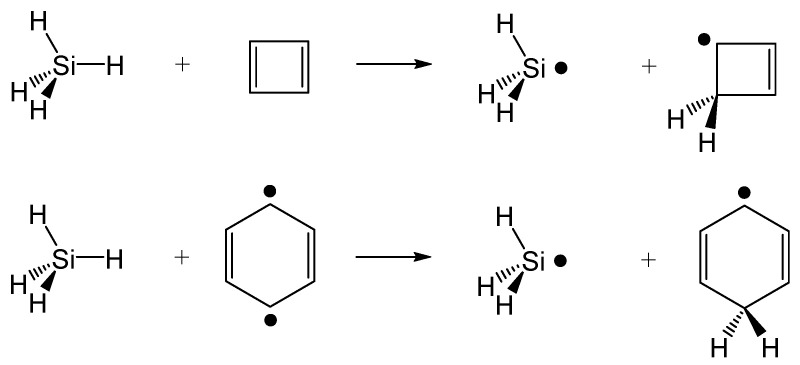
Hydrogen-atom abstraction of SiH4 by cyclobutadiene and *p*-benzyne.

Here, we used a minimal active space of three spin-adapted diabatic states, two for the reactant state corresponding to the closed-shell configuration $\Theta_{\mathrm{CS}}^{R}$ and the open-shell state $\Theta_{\mathrm{OS}}^{R}$ of the diradical species, and one for the product state $\Theta_{\mathrm{HAT}}^{P}$ consisting of two separate radical species that are spin coupled (Figure 5). Each of the diabatic states are expressed in terms of two fragment block-localized determinants

(16) $$\Theta_{\mathrm{CS}}^{R}=N_{\mathrm{CS}}^{20}\{\hat{A}\{(\chi_{\mathrm{DR}}^{\mathrm{core}}\chi_{H}^{\alpha }\chi_{H}^{\beta })(\gamma_{{\mathrm{SiH}}_{4}}^{\mathrm{core}}\gamma_{H}^{\alpha }\gamma_{H}^{\beta })\}]+N_{\mathrm{CS}}^{02}[\hat{A}\{(\chi_{\mathrm{DR}}^{\mathrm{core}}\chi_{L}^{\alpha }\chi_{L}^{\beta })(\gamma_{{\mathrm{SiH}}_{4}}^{\mathrm{core}}\gamma_{H}^{\alpha }\gamma_{H}^{\beta })\}]$$

(17) $$\Theta_{\mathrm{OS}}^{R}=\frac{1}{2}[\hat{A}\{(\chi_{\mathrm{DR}}^{\mathrm{core}}\chi_{H}^{\alpha }\chi_{L}^{\beta })(\gamma_{{\mathrm{SiH}}_{4}}^{\mathrm{core}}\gamma_{H}^{\alpha }\gamma_{H}^{\beta })\}+\hat{A}\{(\chi_{\mathrm{DR}}^{\mathrm{core}}\chi_{H}^{\beta }\chi_{L}^{\alpha })(\gamma_{{\mathrm{SiH}}_{4}}^{\mathrm{core}}\gamma_{H}^{\alpha }\gamma_{H}^{\beta })\}]$$

(18) $$\Theta_{\mathrm{HAT}}^{P}=\frac{1}{2}[\hat{A}\{(\chi_{\mathrm{DRH}}^{\mathrm{core}}\chi_{H}^{2}\chi_{L}^{\alpha })(\gamma_{{\mathrm{SiH}}_{3}}^{\mathrm{core}}\gamma_{H}^{\beta })\}+\hat{A}\{(\chi_{\mathrm{DRH}}^{\mathrm{core}}\chi_{H}^{2}\chi_{L}^{\beta })(\gamma_{{\mathrm{SiH}}_{3}}^{\mathrm{core}}\gamma_{H}^{\alpha })\}]$$

where the molecular fragment blocks of the diradical (DR) and the substrate (SiH_4_) in the reactant state and those of the HAT product (DRH) and the SiH_3_ free radicals (Scheme 1) are grouped in parentheses, the superscript core denotes a product of doubly occupied orbitals that do not change occupation during the reaction, the superscripts α and β specify the electron spin, the orbitals $\chi_{H}$ and $\chi_{L}$ are the highest-occupied and lowest-unoccupied block-localized KS (BLKS) orbitals of the diradical fragment, and $\gamma_{hyd}$ represents the 1 s orbital of the hydrogen atom (it is a BLKS orbital of Si-H bond in the reactant state) [30].

The Hamiltonian matrix functional H[D(r)] is expressed in terms of the BLKS determinant functions as a 6 × 6 matrix. Its elements are determined as follows, keeping in mind that the determinant states are individually optimized to yield the variational diabatic states (VDS) that are best valence-bond-like representations of these Lewis structures [70,71,72,73].

**Figure 6:**
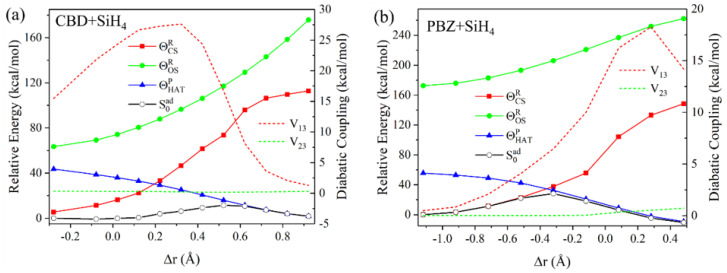
Adiabatic ground-state ($S_{0}^{\mathrm{ad}}$) and diabatic state potential energy profiles for the hydrogen atom abstraction of SiH_4_ by cyclobutadiene (CBD) in (**a**), and by *p*-benzyne (PBZ) in (**b**). The adiabatic potential energies are presented as black curves and those for the reactant diabatic states ($\Theta_{\mathrm{CS}}^{R}$ and $\Theta_{\mathrm{OS}}^{R}$ ) and product diabatic state ($\Theta_{\mathrm{HAT}}^{P}$ ) are shown as colored solid curves. The effective coupling values between the product state and the closed-shell (CS) reactant state (V_13_) and the open-shell (OS) reactant state (V_23_) are shown as dotted curves. The geometries are obtained by migrating the transferring hydrogen atom in the framework of the transition-state structure. The reaction coordinate is defined as $\Delta r=r_{\mathrm{Si}-H}-r_{C-H}$. The M06-HF functional and the cc-pVTZ basis set were used in MSDFT calculations.

- The diagonal elements of H[D(r)] are directly determined as the BLKS-DFT energies of the corresponding determinants. The two reactant diabatic states can be separately obtained as the lower root of a 2 × 2 NOSI diagonalization of the two states in Equations (16) and (17) for illustration in Figure 6, although it is not needed to determine the potential energy surface of the adiabatic ground state. The four determinants in Equations (16) and (17) involve block-local excitations, for which the optimization has been detailed in reference [41]. For the product state, only ground-state BLKS optimization is sufficient;
- The spin-coupling matrix element between the spin-pair determinants in Equation (18) is evaluated using Equations (1) and (5), which requires a separate BLKS calculation of the triplet state with *M_S_* = +1;
- For all other off-diagonal matrix elements of H[D(r)], we used the DFT energy-scaled nonorthogonal determinant value to approximate $H_{AB}[D_{AB}(r)]$ (Equation (6));
- To examine the variations of state interactions as the HAT occurs, we also computed the effective diabatic coupling values between the reactant (Equations (16) and (17)) and product (Equation (18)) states, denoted as $V_{13}$ and $V_{23}$ according to $V_{RP}=H_{RP}-\varepsilon_{g}S_{RP}$, where *R* = 1 and 2, *P* = 3, and $\varepsilon_{g}$ is the adiabatic ground-state energy.

The two processes in Scheme 1 correspond to hydrogen-atom abstractions by a π-type diradical (cyclobutadiene) and a σ-type diradical (*p*-benzyne), and the former has been previously described [1], providing a reference of data for comparison with the present MAS in MSDFT-NOSI calculations.

Shown in Figure 6 are the reaction energy profiles of the adiabatic ground state ($S_{0}^{ad}$) and the diabatic states for the two reactant states, $\Theta_{\mathrm{CS}}^{R}$ and $\Theta_{\mathrm{OS}}^{R}$, along with the HAT state $\Theta_{\mathrm{HAT}}^{P}$. In addition, the effective reactant-product diabatic coupling terms are displayed. Evidently, the open-shell diabatic state (Equation (17)) has the highest energy in both reactions (Scheme 1), and its effective coupling (V_23_) with the product state (dotted green curves) is weak with little variations along the reaction coordinate (Figure 6). On the other hand, interactions between the CS reactant state and product state (V_13_) is strong, reaching its maxima near the transition states for the two HAT reactions. The substantial difference in the effective diabatic couplings between $\Theta_{\mathrm{CS}}^{R}$ and $\Theta_{\mathrm{OS}}^{R}$ diabatic states indicates that the reactivities of both the π and σ types of biradicals in the singlet states are dominantly determined by the closed-shell states. This result is in good accord with previous analyses by Hoffman and coworkers who found that both singlet diradicals are best characterized by two closed-shell configurations [1].

For comparison, the computed energy barrier for the hydrogen-atom abstraction of SiH_4_ by cyclobutadiene is 12 kcal/mol MSDFT-NOSI employing the M06-HF functional and the cc-pVTZ basis set (Figure 6), which is in reasonable accord with a value of 15 kcal/mol using CCSD(T)/cc-pVTZ//B3LYP/cc-pVTZ [1]. Employing the same structures reported by Hoffmann and coworkers [1], we found that the difference in the reaction energy between the two methods is greater (2 vs. −2 kcal/mol) [1]. There is no reported computational study of the HAT between SiH_4_ and *p*-benzyne. We optimized the reaction pathway using M06-HF/cc-pVTZ and obtained an energy barrier of 24 kcal/mol, significantly greater than the reaction by cyclobutadiene, and an energy of reaction of −3 kcal/mol. For comparison, they are, respectively, 28 kcal/mol and −1 kcal/mol from MSDFT-NOSI calculations. Interestingly, the reaction involving the CS diabatic state (Equation (16)) is formally an electron transfer from the σ_Si-H_ bond to the lowest unoccupied BLKS orbital of the diradical reactants. Although both reactions occur dominantly via the charge transfer pathway, it is remarkable to note that through-bond interactions between the two σ-frontier orbitals of *p*-benzyne lead to significant energy separations between the in-phase and out-phase combinations, resulting in a CS diradical character. In the case of cyclobutadiene, on the other hand, the breaking of orbital degeneracy is due to the Jahn–Teller distortion of the molecular geometry. For comparison, the nature of orbital interactions in the OS diabatic state (Equation (17)) follows a spin-exchange mechanism between the SiH_4_ and the diradical configurations (Figure 5).

## 5. Conclusions

In this work, we described the use of a minimal active space of two electrons in two orbitals to treat diradical species using multistate density functional theory (MSDFT). Because dynamic correlation is included in the first place in each basis state function, it is possible to yield quantitative results for these systems that would otherwise require a much larger active space plus correction for dynamic correlation in wave function theory. The optimization of the Hamiltonian matrix density in terms of its total subspace ensemble energy with respect to the multistate matrix density **D**(**r**) can be performed variationally. In the present study, we adopted a nonorthogonal state interaction (NOSI) approach to approximate the self-consistent-field optimization in which both orbitals and state coefficients were simultaneously changed. We note here that since the Slater determinants in the MAS were used to represent the matrix density **D**(**r**) of interacting states of the subspace, the procedure was a state interaction rather than a configuration interaction. In this article, we first illustrated the computational procedure and its performance on the well-understood case of hydrogen molecule dissociation, and then we extended the same MAS (in terms of constrained Kohn–Sham determinant states) to other prototypical diradical cases, including singlet–triplet-energy splitting of benzyne isomers, Jahn–Teller structural tautomerization of cyclobutadiene and polyacenes up to 15 fused rings. The method was also used to investigate the competition between the charge transfer and spin exchange mechanism in the hydrogen abstraction reaction of SiH_4_ by the π-type diradical cyclobutadiene and σ-type diradical *p*-benzyne. In comparison with accurate results, we found that MAS-MSDFT can be used to adequately model the energies and reactivities of the diradicals examined in this work, and we anticipate that this trend can be extended to other diradical species. The overall computational cost was slightly greater than that needed for *N*-sperate KS-DFT calculations, with *N* being the number of determinants in the MAS, plus the time needed to evaluate the nonorthogonal matrix element.

## Figures and Tables

**Table 1:** Computed adiabatic singlet–triplet-energy gaps ($\Delta E_{ST}=E_{S}-E_{T}$ ) of meta-, ortho- and para-benzyne, along with experimental values. Energies are presented in kcal/mol. The cc-pVTZ basis set was used in all calculations, and the M06-HF correlation functional is used in Kohn–Sham and multistate density functional theory (MSDFT) calculations.

Method	*p*-Benzyne	*m*-Benzyne	*o*-Benzyne	MUE
RDFT	41.7	19.3	−37.3	28.7
UDFT	−1.9	−4.0	−37.3	6.4
CASSCF	−0.7	−12.9	−28.5	6.8
CASPT2	−3.6	−19.7	−35.0	1.3
SF-CCSD(UHF) ^a^	−3.9	−19.2	−37.6	0.7
MSDFT	−4.7	−19.1	−36.9	0.5
Exp. ^b^	−3.8	−21.0	−37.5	-

^a^ Reference [51]. ^b^ Reference [52].

## Data Availability

The data that supports the findings of this study are available within the article and its Supplementary Materials. Additional information is available from the corresponding author upon reasonable request.

## Supplementary Materials

The following supporting information can be downloaded at: https://www.mdpi.com/article/10.3390/molecules27113466/s1.

## Sample Availability

Computational input samples are available from the authors.

## References

[1] Stuyver T., Chen B., Zeng T., Geerlings P., De Proft F., Hoffmann R. (2019). Do Diradicals Behave Like Radicals?. Chem. Rev..

[2] Zhang D., Truhlar D.G. (2020). Spin Splitting Energy of Transition Metals: A New, More Affordable Wave Function Benchmark Method and Its Use to Test Density Functional Theory. J. Chem. Theory Comput..

[3] Rajca A. (1994). Organic Diradicals and Polyradicals: From Spin Coupling to Magnetism?. Chem. Rev..

[4] Abe M. (2013). Diradicals. Chem. Rev..

[5] Kaupp G., Teufel E., Hopf H. (1979). First Spectroscopic Detection of Diradicals in photocycloreversions. Angew. Chem. Int. Ed..

[6] Borden W.T., Davidson E.R. (1977). Effects of electron repulsion in conjugated hydrocarbon diradicals. J. Am. Chem. Soc..

[7] Fukui K., Tanaka K. (2006). A Theoretical Study on Biradicals. I. Theoretical Characteristics of Biradicals. Bull. Chem. Soc. Jpn..

[8] Salem L., Rowland C. (1972). The Electronic Properties of Diradicals. Angew. Chem. Int. Ed..

[9] Nakano M. (2017). Electronic Structure of Open-Shell Singlet Molecules: Diradical Character Viewpoint. Top. Curr. Chem..

[10] Scheschkewitz D., Amii H., Gornitzka H., Schoeller W.W., Bourissou D., Bertrand G. (2002). Singlet diradicals: From transition states to crystalline compounds. Science.

[11] Yamaguchi K. (1975). The electronic structures of biradicals in the unrestricted Hartree-Fock approximation. Chem. Phys. Lett..

[12] Nakano M. (2016). Open-Shell-Character-Based Molecular Design Principles: Applications to Nonlinear Optics and Singlet Fission. Chem. Rec..

[13] Rivero P., Jiménez-Hoyos C.A., Scuseria G.E. (2013). Entanglement and Polyradical Character of Polycyclic Aromatic Hydrocarbons Predicted by Projected Hartree–Fock Theory. J. Phys. Chem. B.

[14] Sun Z., Wu J. (2011). Open-shell polycyclic aromatic hydrocarbons. J. Mater. Chem..

[15] Nagai H., Nakano M., Yoneda K., Kishi R., Takahashi H., Shimizu A., Kubo T., Kamada K., Ohta K., Botek E. (2010). Signature of multiradical character in second hyperpolarizabilities of rectangular graphene nanoflakes. Chem. Phys. Lett..

[16] Hajgató B., Szieberth D., Geerlings P., De Proft F., Deleuze M. (2009). A benchmark theoretical study of the electronic ground state and of the singlet-triplet split of benzene and linear acenes. J. Chem. Phys..

[17] Hachmann J., Dorando J.J., Avilés M., Chan G.K.-L. (2007). The radical character of the acenes: A density matrix renormalization group study. J. Chem. Phys..

[18] Cembran A., Song L., Mo Y., Gao J. (2009). Block-Localized Density Functional Theory (BLDFT), Diabatic Coupling, and Their Use in Valence Bond Theory for Representing Reactive Potential Energy Surfaces. J. Chem. Theory Comput..

[19] Ren H., Provorse M.R., Bao P., Qu Z., Gao J. (2016). Multistate Density Functional Theory for Effective Diabatic Electronic Coupling. J. Phys. Chem. Lett..

[20] Gao J., Grofe A., Ren H., Bao P. (2016). Beyond Kohn–Sham Approximation: Hybrid Multistate Wave Function and Density Functional Theory. J. Phys. Chem. Lett..

[21] Mo Y., Gao J., Peyerimhoff S.D. (2000). Energy decomposition analysis of intermolecular interactions using a block-localized wave function approach. J. Chem. Phys..

[22] Mo Y., Bao P., Gao J. (2011). Energy decomposition analysis based on a block-localized wavefunction and multistate density functional theory. Phys. Chem. Chem. Phys..

[23] Liu M., Chen X., Grofe A., Gao J. (2018). Diabatic States at Construction (DAC) through Generalized Singular Value Decomposition. J. Phys. Chem. Lett..

[24] Grofe A., Zhao R., Wildman A., Stetina T.F., Li X., Bao P., Gao J. (2021). Generalization of Block-Localized Wave Function for Constrained Optimization of Excited Determinants. J. Chem. Theory Comput..

[25] Zhao R., Grofe A., Wang Z., Bao P., Chen X., Liu W., Gao J. (2021). Dynamic-then-Static Approach for Core Excitations of Open-Shell Molecules. J. Phys. Chem. Lett..

[26] Grofe A., Chen X., Liu W., Gao J. (2017). Spin-Multiplet Components and Energy Splittings by Multistate Density Functional Theory. J. Phys. Chem. Lett..

[27] Zhao R., Hettich C.P., Chen X., Gao J. (2021). Minimal-active-space multistate density functional theory for excitation energy involving local and charge transfer states. Npj Comput. Mater..

[28] Liu W., Hoffmann M.R. (2014). SDS: The ‘static–dynamic–static’ framework for strongly correlated electrons. Theor. Chem. Acc..

[29] Yang L., Grofe A., Reimers J., Gao J. (2019). Multistate density functional theory applied with 3 unpaired electrons in 3 orbitals: The singdoublet and tripdoublet states of the ethylene cation. Chem. Phys. Lett..

[30] Cembran A., Provorse M.R., Wang C., Wu W., Gao J. (2012). The Third Dimension of a More O’Ferrall-Jencks Diagram for Hydrogen Atom Transfer in the Isoelectronic Hydrogen Exchange Reactions of (PhX)(2)H(*) with X = O, NH, and CH(2). J. Chem. Theory Comput..

[31] Hoffmann R., Swaminathan S., Odell B.G., Gleiter R. (1970). Potential surface for a nonconcerted reaction. Tetramethylene. J. Am. Chem. Soc..

[32] Sirjean B., Glaude P.A., Ruiz-Lopez A.M.F., Fournet R. (2006). Detailed Kinetic Study of the Ring Opening of Cycloalkanes by CBS-QB3 Calculations. J. Phys. Chem. A.

[33] Jaque P., Toro-Labbé A., Geerlings P., De Proft F. (2009). Theoretical Study of the Regioselectivity of [2 + 2] Photocycloaddition Reactions of Acrolein with Olefins. J. Phys. Chem. A.

[34] Di Valentin C., Freccero M., Gandolfi R., Rastelli A. (2000). Concerted vs. Stepwise Mechanism in 1,3-Dipolar Cycloaddition of Nitrone to Ethene, Cyclobutadiene, and Benzocyclobutadiene. A Computational Study. J. Org. Chem..

[35] Bai S., Barbatti M. (2019). Mechanism of Spin-Exchange Internal Conversion: Practical Proxies for Diabatic and Nonadiabatic Couplings. J. Chem. Theory Comput..

[36] Qu Z. (2020). Reactivities of singlet oxygen: Open-shell or closed-shell?. Phys. Chem. Chem. Phys..

[37] Grofe A., Qu Z., Truhlar D.G., Li H., Gao J. (2017). Diabatic-At-Construction Method for Diabatic and Adiabatic Ground and Excited States Based on Multistate Density Functional Theory. J. Chem. Theory Comput..

[38] Frisch M.J., Trucks G., Schlegel H.B., Scuseria G., Robb M., Cheeseman J., Scalmani G., Barone V., Mennucci B., Petersson G. (2009). Gaussian 09, Revision A. 1.

[39] Werner H., Knowles P., Amos R., Bernhardsson A., Berning A., Celani P., Cooper D., Deegan M., Dobbyn A., Eckert F. (2012). MolPro2012.

[40] Dunning T.H. (1989). Gaussian Basis Sets for Use in Correlated Molecular Calculations. I. The Atoms Boron through Neon and Hydrogen. J. Chem. Phys..

[41] Bao P., Hettich C.P., Shi Q., Gao J. (2021). Block-Localized Excitation for Excimer Complex and Diabatic Coupling. J. Chem. Theory Comput..

[42] Schmidt M.W., Baldridge K.K., Boatz J.A., Elbert S., Gordon M.S., Jensen J.H., Koseki S., Matsunaga N., Nguyen K.A., Su S. (1993). General atomic and molecular electronic structure system. J. Comput. Chem..

[43] Nguyen N.A., Csonka G.I., Kolossváry I. (1997). Simple tests for density functional methods. J. Comput. Chem..

[44] Johnson B.G., Gonzales C.A., Gill P.M.W., Pople J.A. (1994). A density functional study of the simplest hydrogen abstraction reaction. Effect of self-interaction correction. Chem. Phys. Lett..

[45] Ziegler T., Rauk A., Baerends E.J. (1977). On the Calculation of Multiplet Energies by the Hartree-Fock-Slater Method. Theor. Chim. Acta.

[46] Clapham S.E., Hadzovic A., Morris R.H. (2004). Mechanisms of the H2-hydrogenation and transfer hydrogenation of polar bonds catalyzed by ruthenium hydride complexes. Coord. Chem. Rev..

[47] Su Y., Wang X., Wang L., Zhang Z., Wang X., Song Y., Power P.P. (2016). Thermally controlling the singlet–triplet energy gap of a diradical in the solid state. Chem. Sci..

[48] Hajgató B., Huzak M., Deleuze M.S. (2011). Focal Point Analysis of the Singlet–Triplet Energy Gap of Octacene and Larger Acenes. J. Phys. Chem. A.

[49] Goldberg A.H., Dougherty D.A. (1983). Effects of through-bond and through-space interactions on singlet-triplet energy gaps in localized biradicals. J. Am. Chem. Soc..

[50] Wei H., Hrovat D.A., Mo Y., Hoffmann R., Borden W.T. (2009). The Contributions of Through-Bond Interactions to the Singlet-Triplet Energy Difference in 1,3-Dehydrobenzene. J. Phys. Chem. A.

[51] Ana-Maria Y.S., Cristian C., Krylov A.I. (2004). Bonding Patterns in Benzene Triradicals from Structural, Spectroscopic, and Thermochemical Perspectives. J. Phys. Chem. A.

[52] Wenthold P.G., Squires R.R., Lineberger W.C. (1998). Ultraviolet Photoelectron Spectroscopy of the o-, m-, and p-Benzyne Negative Ions. Electron Affinities and Singlet−Triplet Splittings for o-, m-, and p-Benzyne. J. Am. Chem. Soc..

[53] Borden W.T., Davidson E.R., Hart P. (1978). The potential surfaces for the lowest singlet and triplet states of cyclobutadiene. J. Am. Chem. Soc..

[54] Wu J.I.-C., Mo Y., Evangelista F.A., Schleyer P.V.R. (2012). Is cyclobutadiene really highly destabilized by antiaromaticity?. Chem. Commun..

[55] Karadakov P.B. (2008). Ground- and Excited-State Aromaticity and Antiaromaticity in Benzene and Cyclobutadiene. J. Phys. Chem. A.

[56] Eckert-Maksić M., Vazdar M., Barbatti M., Lischka H., Maksić Z. (2006). Automerization reaction of cyclobutadiene and its barrier height: An ab initio benchmark multireference average-quadratic coupled cluster study. J. Chem. Phys..

[57] Levchenko S., Krylov A.I. (2004). Equation-of-motion spin-flip coupled-cluster model with single and double substitutions: Theory and application to cyclobutadiene. J. Chem. Phys..

[58] Kollmar H., Staemmler V. (1978). Violation of Hund’s rule by spin polarization in molecules. Theor. Chim. Acta.

[59] Thatcher B.W. (1976). Can a square or effectively square singlet be the ground state of cyclobutadiene. J. Am. Chem. Soc..

[60] Hrovat D.A., Borden W.T. (1997). Violations of Hund’s rule in molecules—Where to look for them and how to identify them. J. Mol. Struc.-Theochem..

[61] Tofanelli M.A., Salorinne K., Ni T.W., Malola S., Newell B., Phillips B., Hakkinen H., Ackerson C.J. (2016). Jahn-Teller Effects in Au25(SR)18. Chem. Sci..

[62] Tachikawa H. (2018). Jahn–Teller Effect of the Benzene Radical Cation: A Direct ab Initio Molecular Dynamics Study. J. Phys. Chem. A.

[63] Tachikawa H., Lund A. (2022). Structures and electronic states of trimer radical cations of coronene: DFT–ESR simulation study. Phys. Chem. Chem. Phys..

[64] Senn F., Krykunov M. (2015). Excited State Studies of Polyacenes Using the All-Order Constricted Variational Density Functional Theory with Orbital Relaxation. J. Phys. Chem. A.

[65] Bendikov M., Duong H.M., Starkey K., Houk K.N., Carter A.E.A., Wudl F. (2004). Oligoacenes: Theoretical Prediction of Open-Shell Singlet Diradical Ground States. J. Am. Chem. Soc..

[66] Qu Z., Zhang D., Liu C., Jiang Y. (2009). Open-Shell Ground State of Polyacenes: A Valence Bond Study. J. Phys. Chem. A.

[67] Yang Y., Davidson E.R., Yang W. (2016). Nature of ground and electronic excited states of higher acenes. Proc. Natl. Acad. Sci. USA.

[68] Houk K.N., Lee P.S., Nendel M. (2001). Polyacene and Cyclacene Geometries and Electronic Structures: Bond Equalization, Vanishing Band Gaps, and Triplet Ground States Contrast with Polyacetylene. J. Org. Chem..

[69] Hariharan P.C., Pople J.A. (1973). The influence of polarization functions on molecular orbital hydrogenation energies. Theor. Chim. Acta.

[70] Cooper D.L., Gerratt J., Raimondi M. (1991). Applications of spin-coupled valence bond theory. Chem. Rev..

[71] Mo Y., Gao J. (2000). An Ab Initio Molecular Orbital−Valence Bond (MOVB) Method for Simulating Chemical Reactions in Solution. J. Phys. Chem. A.

[72] Song L., Mo Y., Gao J. (2009). An Effective Hamiltonian Molecular Orbital-Valence Bond (MOVB) Approach for Chemical Reactions as Applied to the Nucleophilic Substitution Reaction of Hydrosulfide Ion and Chloromethane. J. Chem. Theory Comput..

[73] Song L., Gao J. (2008). On the Construction of Diabatic and Adiabatic Potential Energy Surfaces Based on Ab Initio Valence Bond Theory. J. Phys. Chem. A.

