# Supplementary material for: Minimal Active Space for Diradicals Using Multistate Density Functional Theory
Source: Molecules. 2022 May 27;27(11):3466. doi: 10.3390/molecules27113466 (PMC9182067; doi:10.3390/molecules27113466)
Supplement: Supplementary file 1 [file molecules-27-03466-s001.zip › molecules-1718531-supplementary.pdf]

Minimal Active Space for Diradicals using Multistate Density Functional Theory

Jingting Han,[1] Ruoqi Zhao,[1,2] Yujie Guo,[1] Zexing Qu,[1*] and Jiali Gao,[2,3*]

[1]Institute of Theoretical Chemistry, College of Chemistry, Jilin University, Changchun, 130023, China

[2]Institute of Systems and Physical Biology, Shenzhen Bay Laboratory, Shenzhen 518055, China;

[3]Department of Chemistry and Supercomputing Institute, University of Minnesota, Minneapolis, MN 55455, USA;

*Corresponding author e-mail:

zxqu@jlu.edu.cn

gao@jialigao.org

Contents:

Cartesian Coordinates
Supporting Table S1-S11

Cartesian Coordinates

m-benzyne-singlet

C	0.00000000	0.00000000	1.52434876
C	0.00000000	1.11400387	0.71807243
C	0.00000000	1.19541590	-0.64924316
C	0.00000000	0.00000000	-1.36455522
C	0.00000000	-1.19541590	-0.64924316
C	0.00000000	-1.11400387	0.71807243
H	0.00000000	0.00000000	2.60049893
H	0.00000000	2.15061382	-1.15152071
H	0.00000000	0.00000000	-2.44513463
H	0.00000000	-2.15061382	-1.15152071

m-benzyne-triplet

C	0.00000000	0.00000000	1.47476057

C	0.00000000	1.16081494	0.73771268
C	0.00000000	1.21480572	-0.63425815
C	0.00000000	0.00000000	-1.31843500
C	0.00000000	-1.21480572	-0.63425815
C	0.00000000	-1.16081494	0.73771268
H	0.00000000	0.00000000	2.55577357
H	0.00000000	2.15219144	-1.16834180
H	0.00000000	0.00000000	-2.39849777
H	0.00000000	-2.15219144	-1.16834180

o-benzyne-singlet

C	0.00000000	0.61367532	1.22825479
C	0.00000000	1.45494224	0.12962706
C	0.00000000	0.70288043	-1.04792310
C	0.00000000	-0.70288043	-1.04792310
C	0.00000000	-1.45494224	0.12962706
C	0.00000000	-0.61367532	1.22825479
H	0.00000000	2.53149585	0.13611206
H	0.00000000	1.22098935	-1.99586458
H	0.00000000	-1.22098935	-1.99586458
H	0.00000000	-2.53149585	0.13611206

o-benzyne-triplet

C	0.00000000	0.69619542	1.28594736
C	0.00000000	1.39879107	0.11220023
C	0.00000000	0.69125870	-1.09359221
C	0.00000000	-0.69125870	-1.09359221
C	0.00000000	-1.39879107	0.11220023
C	0.00000000	-0.69619542	1.28594736
H	0.00000000	2.47957669	0.12141115
H	0.00000000	1.23092248	-2.02830853
H	0.00000000	-1.23092248	-2.02830853
H	0.00000000	-2.47957669	0.12141115

p-benzyne-singlet

C	1.21747933	0.70133043	0.00000000
C	0.00000000	1.32991457	0.00000000
C	-1.21747933	0.70133043	0.00000000
C	-1.21747933	-0.70133043	0.00000000
C	0.00000000	-1.32991457	0.00000000
C	1.21747933	-0.70133043	0.00000000
H	2.14781426	1.24892719	0.00000000
H	-2.14781426	1.24892719	0.00000000
H	-2.14781426	-1.24892719	0.00000000

H	2.14781426	-1.24892719	0.00000000

p-benzyne-triplet

C	1.22340653	0.69851451	0.00000000
C	0.00000000	1.32103074	0.00000000
C	-1.22340653	0.69851451	0.00000000
C	-1.22340653	-0.69851451	0.00000000
C	0.00000000	-1.32103074	0.00000000
C	1.22340653	-0.69851451	0.00000000
H	2.14705135	1.25796605	0.00000000
H	-2.14705135	1.25796605	0.00000000
H	-2.14705135	-1.25796605	0.00000000
H	2.14705135	-1.25796605	0.00000000

Reactant of reaction of Aryne+SiH4

C	0.01755370	-0.74268239	1.22244805
C	0.03088509	-0.11257062	0.00000000
C	0.01755370	-0.74268239	-1.22244805
C	-0.00953034	-2.13962713	-1.22326103
C	-0.02139264	-2.76213582	0.00000000
C	-0.00953034	-2.13962713	1.22326103
H	0.02745804	-0.18437138	2.14775094
H	0.02745804	-0.18437138	-2.14775094
H	-0.02095225	-2.69901636	-2.14706418
H	-0.02095225	-2.69901636	2.14706418
H	0.05718528	1.78724734	0.00000000
Si	-0.00891196	3.20562196	0.00000000
H	-0.72853765	3.65065854	1.20691805
H	1.36001049	3.75101022	0.00000000
H	-0.72853765	3.65065854	-1.20691805

Product of reaction of Aryne+SiH4

C	0.01755370	-0.74268239	1.22244805
C	0.03088509	-0.11257062	0.00000000
C	0.01755370	-0.74268239	-1.22244805
C	-0.00953034	-2.13962713	-1.22326103
C	-0.02139264	-2.76213582	0.00000000
C	-0.00953034	-2.13962713	1.22326103
H	0.02745804	-0.18437138	2.14775094
H	0.02745804	-0.18437138	-2.14775094
H	-0.02095225	-2.69901636	-2.14706418
H	-0.02095225	-2.69901636	2.14706418
H	0.04611152	0.98732399	0.00000000
Si	-0.00891196	3.20562196	0.00000000

H	-0.72853765	3.65065854	1.20691805
H	1.36001049	3.75101022	0.00000000
H	-0.72853765	3.65065854	-1.20691805

Reactant of reaction of CBD+SiH4

C	0.87238623	-0.70074239	0.00000000
C	0.07991465	-1.44640156	1.01741326
C	-0.68111997	-2.06750183	0.00000000
C	0.07991465	-1.44640156	-1.01741326
H	-1.52092886	-2.74423765	0.00000000
H	1.94864348	-0.57416443	0.00000000
H	0.07805860	-1.47028036	-2.09404769
H	0.07805860	-1.47028036	2.09404769
H	0.36359021	0.92133240	0.00000000
Si	-0.15260539	2.24777016	0.00000000
H	0.31304001	2.96633661	1.20863130
H	0.31304001	2.96633661	-1.20863130
H	-1.63186557	2.18385522	0.00000000

Product of reaction of CBD+SiH4

C	0.87238623	-0.70074239	0.00000000
C	0.07991465	-1.44640156	1.01741326
C	-0.68111997	-2.06750183	0.00000000
C	0.07991465	-1.44640156	-1.01741326
H	-1.52092886	-2.74423765	0.00000000
H	1.94864348	-0.57416443	0.00000000
H	0.07805860	-1.47028036	-2.09404769
H	0.07805860	-1.47028036	2.09404769
H	0.54316529	0.34883542	0.00000000
Si	-0.15260539	2.24777016	0.00000000
H	0.31304001	2.96633661	1.20863130
H	0.31304001	2.96633661	-1.20863130
H	-1.63186557	2.18385522	0.00000000

Supporting Table S1

Table S1: The absolute energies (in a.u.) for the lowest singlet and triplet states, S_0 and T_1, and the diradical index of $N_D/2$ for H_2 molecule as a function of H-H bond length. The CASSCF, (CAS) Restricted Hartree-Fock (RHF) and Unrestricted Hartree-Fock (UHF) methods are used at cc-pVQZ level respectively.

R_{H-H}(Å)	S_0^{CAS}	T_1^{CAS}	S_0^{RHF}	S_0^{UHF}	$N_D^{UHF}/2$	$N_D^{CAS}/2$
0.5	-1.06251	-0.56367	-1.06469	-1.06469	0	0.021839
0.55	-1.09555	-0.62469	-1.09641	-1.09641	0	0.025772

0.57	-1.10524	-0.64546	-1.10553	-1.10553	0	0.027528
0.59	-1.11332	-0.66453	-1.11301	-1.11301	0	0.029381
0.6	-1.11681	-0.6735	-1.11619	-1.11619	0	0.030346
0.61	-1.11997	-0.68212	-1.11904	-1.11904	0	0.03135
0.63	-1.1254	-0.69839	-1.12381	-1.12381	0	0.033455
0.65	-1.12974	-0.7135	-1.12747	-1.12747	0	0.035676
0.7	-1.13673	-0.74699	-1.13262	-1.13262	0	0.041814
0.75	-1.13943	-0.77556	-1.13331	-1.13331	0	0.048907
0.8	-1.13905	-0.80028	-1.13075	-1.13075	0	0.057023
0.9	-1.13221	-0.84106	-1.11914	-1.11914	0	0.076844
1	-1.12073	-0.87326	-1.10246	-1.10246	0	0.102282
1.2	-1.09303	-0.91974	-1.06327	-1.06327	0	0.173937
1.4	-1.06638	-0.94976	-1.02359	-1.03228	0.428482	0.277534
1.6	-1.04448	-0.96901	-0.98694	-1.0164	0.685476	0.41033
1.8	-1.02819	-0.9812	-0.95437	-1.0083	0.825784	0.556844
2	-1.01702	-0.98879	-0.92594	-1.00418	0.903046	0.693992
2.2	-1.00988	-0.99342	-0.90138	-1.00208	0.945742	0.803804
2.4	-1.00555	-0.99619	-0.88025	-1.00101	0.969699	0.881113
2.6	-1.00303	-0.9978	-0.86214	-1.00046	0.98321	0.930699
3	-1.00082	-0.99925	-0.83333	-1.00004	0.994786	0.97796
3.5	-1.00008	-0.99975	-0.80784	-0.99992	0.998806	0.995038
4	-0.99993	-0.99986	-0.79038	-0.9999	0.9997	0.99892
5	-0.99989	-0.99989	-0.76966	-0.99989	0.999924	0.999953

Table S2: The absolute energies (in a.u.) for the adiabatic lowest singlet and triplet states, S_0 and T_1, and diabatic components of Θ_{CS}^{02}, Θ_{CS}^{20} and Θ_{OS}^{S} of H_2 molecule computed with MOVB. All the calculations were performed at cc-pVQZ level. $R_{H\text{-}H}$ is the bond length of H2.

$R_{H\text{-}H}$(Å)	S_0^{MOVB}	T_1^{MOVB}	Θ_{OS}^{S}	Θ_{CS}^{02}	Θ_{CS}^{20}	S_0^{FCI}	T_1^{FCI}
0.5	-1.06771	-0.56496	-0.51601	0.340175	-1.06471	-1.10338	-0.57849
0.55	-1.10012	-0.62319	-0.56689	0.229438	-1.09642	-1.13527	-0.63864
0.57	-1.10955	-0.64282	-0.58337	0.189699	-1.10555	-1.14449	-0.65907
0.59	-1.11735	-0.66076	-0.59803	0.152176	-1.11302	-1.15207	-0.67782
0.6	-1.1207	-0.66915	-0.60475	0.134178	-1.1162	-1.15531	-0.68663
0.61	-1.12373	-0.6772	-0.61109	0.116659	-1.11905	-1.15822	-0.69508
0.63	-1.12886	-0.69234	-0.62271	0.082969	-1.12382	-1.16313	-0.71104
0.65	-1.13291	-0.70632	-0.63306	0.050951	-1.12748	-1.16694	-0.72585
0.7	-1.1391	-0.73708	-0.65421	-0.02263	-1.13263	-1.17253	-0.75860
0.75	-1.14097	-0.76309	-0.66992	-0.0883	-1.13332	-1.17375	-0.78647
0.8	-1.13971	-0.78555	-0.68148	-0.14732	-1.13076	-1.17182	-0.81055
0.9	-1.13109	-0.82282	-0.69586	-0.24907	-1.11915	-1.16178	-0.85015

1	-1.11789	-0.85288	-0.70276	-0.33332	-1.10247	-1.14710	-0.88131
1.2	-1.08716	-0.89803	-0.70561	-0.46257	-1.06328	-1.11341	-0.92605
1.4	-1.05794	-0.92806	-0.70254	-0.55385	-1.02361	-1.08149	-0.95463
1.6	-1.03366	-0.947	-0.69709	-0.61869	-0.98697	-1.05512	-0.97264
1.8	-1.01504	-0.95833	-0.69002	-0.6649	-0.95441	-1.03525	-0.98377
2	-1.00157	-0.96478	-0.68168	-0.69784	-0.926	-1.02144	-0.99053
2.2	-0.99224	-0.96826	-0.67234	-0.72128	-0.90146	-1.01251	-0.99455
2.4	-0.98595	-0.97003	-0.66232	-0.73788	-0.88037	-1.00706	-0.99690
2.6	-0.98179	-0.97091	-0.65195	-0.74953	-0.86228	-1.00389	-0.99825
3	-0.97724	-0.97168	-0.63132	-0.76295	-0.83356	-1.00109	-0.99943
3.5	-0.97503	-0.97242	-0.60777	-0.76906	-0.80814	-1.00016	-0.99981
4	-0.97437	-0.9732	-0.58815	-0.76944	-0.79072	-0.99996	-0.99989
5	-0.9743	-0.97415	-0.5601	-0.76417	-0.76996	-0.99990	-0.99990

Table S3: The absolute energies (in a.u.) for the adiabatic lowest singlet and triplet states, S_0 and T_1, and diabatic components of Θ_{CS}^{02}, Θ_{CS}^{20} and Θ_{OS}^{S} of H_2 molecule computed with MSDFT. All the calculations were performed at cc-pVQZ level and the M06-HF functional was used in MSDFT calculations. $R_{H\text{-}H}$ is the bond length of H_2.

$R_{H\text{-}H}$(Å)	S_0^{MSDFT}	T_1^{MSDFT}	Θ_{OS}^{S}	Θ_{CS}^{02}	Θ_{CS}^{20}
0.5	-1.10445	-0.56741	-0.51357	0.295247	-1.10204
0.55	-1.13674	-0.626	-0.56407	0.183206	-1.13395
0.57	-1.14608	-0.64579	-0.5804	0.143119	-1.14312
0.59	-1.15378	-0.66389	-0.5949	0.105296	-1.15062
0.6	-1.15709	-0.67237	-0.60153	0.087135	-1.15382
0.61	-1.16006	-0.68051	-0.60778	0.069434	-1.15668
0.63	-1.1651	-0.69582	-0.61923	0.035348	-1.16147
0.65	-1.16906	-0.70998	-0.6294	0.002902	-1.16515
0.7	-1.17502	-0.74122	-0.65007	-0.07185	-1.17036
0.75	-1.1766	-0.76775	-0.66526	-0.1388	-1.17111
0.8	-1.17497	-0.79075	-0.67628	-0.19912	-1.16859
0.9	-1.16536	-0.82916	-0.68952	-0.30301	-1.15695
1	-1.1512	-0.86039	-0.69526	-0.38881	-1.14034
1.2	-1.11978	-0.90766	-0.69598	-0.52027	-1.10256
1.4	-1.09081	-0.93933	-0.69127	-0.61281	-1.0654
1.6	-1.06598	-0.95949	-0.68459	-0.6784	-1.03084
1.8	-1.04673	-0.97175	-0.67661	-0.7251	-1.00001
2	-1.03338	-0.97894	-0.66752	-0.75844	-0.97349
2.2	-1.02451	-0.98305	-0.65754	-0.78219	-0.95109
2.4	-1.01858	-0.98542	-0.64693	-0.79901	-0.932
2.6	-1.01459	-0.98686	-0.63601	-0.81075	-0.91563
3	-1.00942	-0.9887	-0.6143	-0.82401	-0.88931

3.5	-1.0061	-0.99065	-0.58954	-0.82985	-0.86514
4	-1.00633	-0.99246	-0.5689	-0.83012	-0.84925
5	-1.00489	-0.99486	-0.5394	-0.82484	-0.83053

Table S4: The absolute energies for singlet and triplet energies in a.u. of p-, m- and o-benzynes computed with restricted DFT (RDFT), unrestricted DFT (UDFT), CASSCF, CASPT2 and MSDFT at cc-pVTZ level. The M06-HF functionals were used in all DFT calculations.

Method	p-benzyne		m-benzyne		o-benzyne	
	singlet	triplet	singlet	triplet	singlet	triplet
RDFT	-230.8387	-230.9052	-230.8741	-230.9048	-230.9551	-230.8956
UDFT	-230.9082	-230.9052	-230.9112	-230.9048	-230.9551	-230.8956
CASSCF	-229.4718	-229.4707	-229.4889	-229.4684	-229.5095	-229.4642
CASPT2	-230.3694	-230.3637	-230.3921	-230.3606	-230.4104	-230.3547
MSDFT	-230.9529	-230.9454	-230.9628	-230.9324	-230.9629	-230.9041

Table S5: The S^2 values for singlet and triplet states of p-, m- and o- benzynes computed with unrestricted M06-HF at cc-pVTZ level.

	singlet	triplet
p-benzyne	1.019761	2.010173
m-benzyne	0.855802	2.034446
o-benzyne	0.0	2.013161

Table S6: The absolute energies for the lowest singlet S_0 and triplet T_1 states of CBD along the interpolation coordinate (IC) from the square (D_{4h}) geometry to the rectangular (D_{4h}) geometry computed with CASSCF(2,2), CASSCF(4,4), MRCI(2,2), MRCI(4,4) and MSDFT at cc-pVTZ level. The M06-HF functional was used in MSDFT calculations.

IC	S_0CAS(2,2)	T_1CAS(2,2)	S_0CAS(4,4)	T_1CAS(4,4)	S_0MRCI(2,2)	T_1MRCI(2,2)
-7	-153.70283	-153.62312	-153.75398	-153.66307	-154.35379	-154.27013
-6	-153.70581	-153.64236	-153.75887	-153.68158	-154.35765	-154.2882
-5	-153.70616	-153.65869	-153.76118	-153.69725	-154.35893	-154.3035
-4	-153.70408	-153.67216	-153.7611	-153.71014	-154.35782	-154.31607
-3	-153.69991	-153.68279	-153.75894	-153.72026	-154.35464	-154.32593

-2	-153.69432	-153.69056	-153.7553	-153.7276	-154.35002	-154.33307
-1	-153.6888	-153.69542	-153.75147	-153.7321	-154.34534	-154.33743
0	-153.68634	-153.69728	-153.74966	-153.73367	-154.34317	-154.33892
1	-153.6888	-153.69542	-153.75147	-153.7321	-154.34534	-154.33743
2	-153.69432	-153.69056	-153.7553	-153.7276	-154.35002	-154.33307
3	-153.69991	-153.68279	-153.75894	-153.72026	-154.35464	-154.32593
4	-153.70408	-153.67216	-153.7611	-153.71014	-154.35782	-154.31607
5	-153.70616	-153.65869	-153.76118	-153.69725	-154.35893	-154.3035
6	-153.70581	-153.64236	-153.75887	-153.68158	-154.35765	-154.2882
7	-153.70283	-153.62312	-153.75398	-153.66307	-154.35379	-154.27013

X	S_0MRCI(4,4)	T_1MRCI(4,4)	S_0MSDFT	T_1MSDFT
-7	-154.36199	-154.27776	-154.71576	-154.61348
-6	-154.36627	-154.29558	-154.7191	-154.6316
-5	-154.36799	-154.31067	-154.72003	-154.64696
-4	-154.36732	-154.32306	-154.71882	-154.65962
-3	-154.36456	-154.33277	-154.71586	-154.66958
-2	-154.36028	-154.33978	-154.71204	-154.67684
-1	-154.35582	-154.34404	-154.70849	-154.68133
0	-154.35365	-154.34547	-154.70703	-154.68296
1	-154.35582	-154.34404	-154.70849	-154.68133
2	-154.36028	-154.33978	-154.71204	-154.67684
3	-154.36456	-154.33277	-154.71586	-154.66958
4	-154.36732	-154.32306	-154.71882	-154.65962
5	-154.36799	-154.31067	-154.72003	-154.64696
6	-154.36627	-154.29558	-154.7191	-154.6316
7	-154.36199	-154.27776	-154.71576	-154.61348

Table S7: The absolute energies for the MSDFT diabatic basis states, E(20) and E(02), and diabatic coupling, V12, for CBD along the interpolation coordinate (IC) from the square (D_{4h}) geometry to the rectangular (D_{4h}) geometry computed with MSDFT at cc-pVTZ level using M06-HF functional. The unite is Hartree.

	E(20)	E(02)	V12
-7	-154.707567	-154.476543	0.044281
-6	-154.709582	-154.511122	0.044486
-5	-154.708774	-154.542960	0.044649
-4	-154.705170	-154.572126	0.044755
-3	-154.698764	-154.598653	0.044769
-2	-154.689523	-154.622542	0.044890
-1	-154.677374	-154.643749	0.044886

0	-154.662195	-154.662195	0.044835
1	-154.677374	-154.643749	0.044886
2	-154.689523	-154.622542	0.044890
3	-154.698764	-154.598653	0.044769
4	-154.705170	-154.572126	0.044755
5	-154.708774	-154.542960	0.044649
6	-154.709582	-154.511122	0.044486
7	-154.707567	-154.476543	0.044281

Table S8: The absolute singlet and triplet energies (in a.u.) for polyacenes computed with UDFT, RDFT and MSDFT at 6-31G(d) level. The B3LYP functional was used in all the DFT calculations.

n	UDFT		RDFT		MSDFT	
	singlet	triplet	singlet	triplet	singlet	triplet
3	-539.53049	-539.45198	-539.53049	-539.45198	-539.39946	-539.30653
4	-693.16576	-693.11201	-693.16576	-693.11201	-692.99861	-692.92897
5	-846.79987	-846.76337	-846.79987	-846.76337	-846.59610	-846.54482
6	-1000.4336	-1000.4110	-1000.4332	-1000.4110	-1000.1919	-1000.1538
7	-1154.0693	-1154.0559	-1154.0654	-1154.0559	-1153.7862	-1153.7595
8	-1307.7061	-1307.6962	-1307.6976	-1307.6962	-1307.3807	-1307.3619
9	-1461.3431	-1461.3341	-1461.3301	-1461.3341	-1460.9757	-1460.9624
10	-1614.9800	-1614.9707	-1614.9628	-1614.9707	-1614.5710	-1614.5617
11	-1768.6167	-1768.6062	-1768.5956	-1768.6062	-1768.1662	-1768.1599
12	-1922.2534	-1922.2534	-1922.2285	-1922.2534	-1921.7615	-1921.7571
13	-2075.8900	-2075.8900	-2075.8619	-2075.8900		
14	-2229.5266	-2229.5266	-2229.4963	-2229.5266		
15	-2383.1632	-2383.1632	-2383.1314	-2383.1632		

Table S9: The Configuration Interaction (CI) matrix for polyacenes with n=3, 6, 9. The index 1 and 2 represent the ^{1}(02) and ^{1}(20) configurations, respectively. S_{ij} and H_{ij} are defined as $S_{ij} = \langle \Psi_i | \Psi_j \rangle$ and $H_{ij} = \langle \Psi_i | H | \Psi_j \rangle$, respectively.

n	i	j	S_{ij}	H_{ij}
3	1	1	1.0000000000	-539.1303853916
	1	2	0.0000000021	0.0410750530
	2	1	0.0000000021	0.0410750530
	2	2	1.0000000000	-539.3931931229
6	1	1	1.0000000000	-1000.0527685650
	1	2	0.0000007080	0.0345434530
	2	1	0.0000007080	0.0345434530
	2	2	1.0000000000	-1000.1829248854
9	1	1	1.0000000000	-1460.8961646354
	1	2	0.0000003006	0.0352278360
	2	1	0.0000003006	0.0352278360
	2	2	1.0000000000	-1460.9596816107

Table S10: The absolute energies for the diabatic reactant ($^1R_{CS}$ and $^1R_{OS}$), product (^{1}P) and the adiabatic ground state (S_0^{ad}) as well as the diabatic couplings (V_{12}^{CS} and V_{12}^{OS}) for (a)CBD+SiH_4 and (b) Aryne+SiH_4 reactions. The reaction coordinates were defined as $\Delta r = r_{Si-H} - r_{C-H}$. All the calculations were performed using MSDFT with M06-HF functional at cc-pVTZ level.

(a)

Δr	$^1R_{CS}$	$^1R_{OS}$	^{1}P	S_0^{ad}	V_{12}^{CS}	V_{12}^{OS}
0.27841	-446.51816	-446.42587	-446.45746	-446.52692	0.0246690	-0.0005288
0.07841	-446.50886	-446.41661	-446.46532	-446.52810	0.0347530	0.0005069
-0.02159	-446.50096	-446.40862	-446.46965	-446.52710	-0.0387565	-0.0004766
-0.12159	-446.49124	-446.39862	-446.47448	-446.52615	0.0424699	0.0004357
-0.22159	-446.47408	-446.38680	-446.48012	-446.52067	0.0434575	-0.0004050
-0.32159	-446.45260	-446.37316	-446.48671	-446.51687	0.0440206	0.0003542
-0.42159	-446.42894	-446.35769	-446.49402	-446.51213	-0.0388024	-0.0002740

-0.52159	-446.40937	-446.34034	-446.50151	-446.50863	-0.0265885	-0.0002580
-0.62159	-446.37399	-446.32085	-446.50868	-446.50991	-0.0129428	0.0002956
-0.72159	-446.35744	-446.29896	-446.51510	-446.51531	-0.0057487	-0.0003556
-0.82159	-446.35198	-446.27435	-446.52047	-446.52054	0.0033011	0.0004105
-0.92159	-446.24677	-446.24677	-446.52406	-446.52408	0.0020393	0.0004933

(b)

Δr	$^1R_{CS}$	$^1R_{OS}$	^1P	S_0^{ad}	V_{12}^{CS}	V_{12}^{OS}
-1.11843	-522.78946	-522.51443	-522.70081	-522.78947	7.6227E-04	1.1124E-06
-0.91843	-522.78408	-522.50929	-522.70497	-522.78410	1.3318E-03	2.6536E-06
-0.71843	-522.77141	-522.49811	-522.71127	-522.77159	3.3145E-03	1.5741E-06
-0.51843	-522.75285	-522.48175	-522.72183	-522.75415	6.4925E-03	2.7966E-06
-0.31843	-522.72936	-522.46123	-522.73749	-522.74450	1.0308E-02	5.1940E-06
-0.11843	-522.70060	-522.43750	-522.75610	-522.76035	1.5927E-02	6.7575E-05
0.08157	-522.62321	-522.41203	-522.77514	-522.77941	2.5840E-02	5.1569E-04
0.28157	-522.57742	-522.38801	-522.79281	-522.79668	2.9132E-02	8.2527E-04
0.48157	-522.55283	-522.37157	-522.80408	-522.80610	2.2609E-02	1.1395E-03

Table S11: The Configuration Interaction (CI) matrix for CBD+SiH_4 and Aryne+SiH_4 reactions. The index 1, 2 and 3 represent the $^1R_{CS}$, $^1R_{OS}$ and ^1P respectively. S_{ij} and H_{ij} are defined as $S_{ij} = \langle \Psi_i | \Psi_j \rangle$ and $H_{ij} = \langle \Psi_i | H | \Psi_j \rangle$, respectively.

Index		CBD+SiH_4		Aryne+SiH_4	
		$\Delta r = 0.82159$		$\Delta r = 0.48157$	
i	j	S_{ij}	H_{ij}	S_{ij}	H_{ij}
1	1	1.000000	-446.351976	1.000000	-522.552826
1	2	-0.453347	202.362674	-0.310347	162.156724
1	3	-0.033349	14.894379	-0.156533	81.848999

2	1	-0.453347	202.362674	-0.310347	162.156724
2	2	1.000000	-446.274354	1.000000	-522.371573
2	3	-0.011086	4.950602	-0.339720	177.618340
3	1	-0.033349	14.894379	-0.156533	81.848999
3	2	-0.011086	4.950602	-0.339720	177.618340
3	3	1.000000	-446.520474	1.000000	-522.804081
